# Intestinal Infarction Caused by Thrombophlebitis of the Portomesenteric Veins as a Complication of Acute Gangrenous Appendicitis After Appendectomy: A Case Report

**DOI:** 10.1097/MD.0000000000001033

**Published:** 2016-06-19

**Authors:** Rui Tang, Xiaodong Tian, Xuehai Xie, Yinmo Yang

**Affiliations:** From the Department of Hepatopancreatobiliary Surgery, Beijing Tsinghua Changgung Hospital, Medical Center, Tsinghua University (RT); and Department of General Surgery, Peking University First Hospital, Beijing, People's Republic of China (XT, XX, YY).

## Abstract

The clinical symptoms of pylephlebitis caused by acute appendicitis are varied and atypical, which leads to delayed diagnosis and poor outcomes. Here, we report a case of intestinal necrosis caused by thrombophlebitis of the portomesenteric veins as a complication of acute appendicitis after appendectomy.

The patient had acute abdominal pain with tenderness and melena on the 3rd day after appendectomy for the treatment of gangrenous appendicitis. He was diagnosed with intestinal infarction caused by thrombophlebitis of the portomesenteric veins based on enhanced CT and diagnostic abdominal paracentesis. The patient was treated by bowel excision anastomosis and thrombectomy. After postoperative antibiotic and anticoagulation treatments, the patient recovered well and was discharged 22 days after the 2nd operation. A follow-up CT scan showed no recurrence of portomesenteric veins thrombosis 3 months later.

Thrombophlebitis of the portomesenteric veins is a rare but fatal complication of acute appendicitis. For all the cases with acute abdominal pain, the possibility of thrombophlebitis should be considered as a differential diagnosis. Once pylephlebitis is suspected, enhanced CT scan is helpful for early diagnosis, and sufficient control of inflammation as well as anticoagulant therapy should be performed.

## INTRODUCTION

Thrombophlebitis of the portomesenteric veins is a kind of pylephlebitis which is normally associated with intraperitoneal septic conditions, including acute appendicitis, colonic diverticulitis, and cholangitis.^1^ With the advances in antibiotic therapy, the incidence of portomesenteric veins thrombosis as a complication of acute appendicitis has become very low. However, the mortality rate remains high, due to the nonspecific symptoms and low index of suspicion. This disease is difficult to be diagnosed in the early period, and frequently leads to the progression of intestinal necrosis, liver abscess, and septic shock.

In this report, we presented a case of intestinal infarction caused by thrombophlebitis of the portomesenteric veins after appendectomy. The patient was successfully treated by surgical procedure with bowel excision anastomosis and thrombectomy as well as the intravenous (IV) antibiotics and anticoagulation therapy.

## CASE REPORT

**FIGURE 1:**
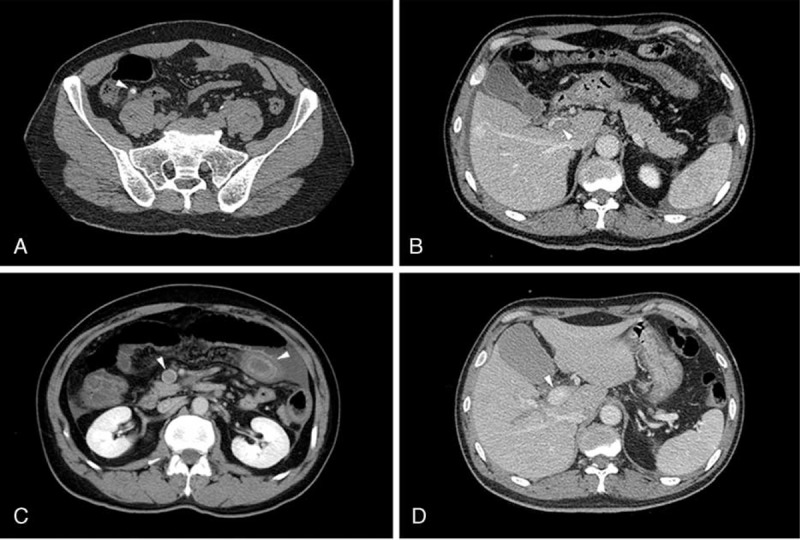
(A) An enlarged and thick-walled appendix with fecalith inside (arrows) at the level of the lower abdomen. Enhanced CT scan indicated visible thrombus with defects in the lumen of PV (B) and SMV (C), the thickened wall of small intestine indicating ischemia (C). (D) The portomesenteric veins thrombosis diminished 3 months after treatment. CT = computed tomography; PV = portal vein; SMV = superior mesenteric vein.

A 56-year-old man visited our emergency department primarily due to right lower quadrant abdominal pain and chills fever for 1 day. He had no previous medical history. The maximum temperature (T_max_) was 38.9°C, and the examination of the abdomen revealed a local tenderness and rebound with guarding at Mcburney's point. Laboratory tests showed white blood cell (WBC) count 20.93 × 10^9^/L, neutrophils were 88.0%. The results of blood coagulation screening were in normal scales: prothrombin time 13.80 s, prothrombin activity 65%, and fibrin degradation product 3.9 mg/L. No liver or kidney dysfunction. Electrocardiograph was normal. Abdominal ultrasonography and simple multislice computed tomography (MSCT) scan of the abdomen and pelvic cavity indicated an acute appendicitis with appendicolith, and no other abnormality was found (Figure 1A). The patient was admitted to the hospital and received laparotomy appendectomy. Approval was obtained from the hospital's ethics committee, and patient consent was obtained.

**FIGURE 2:**
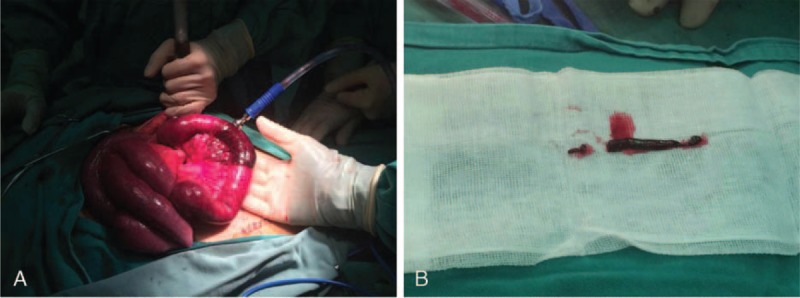
(A) Intestine necrosis was observed during the operation. (B) Septic thrombus was removed by a Fogarty catheter in the portal vein.

During the operation, a gangrenous appendix with perforation at the base was resected, and no abnormalities were found. IV infusion of sulperazone (3.0 g, q12h) was performed for 2 days and then moxifloxacin (0.4 g, qd) was administrated orally. The patient had slightly improved abdominal pain but his body temperature was still high as 38.2°C, WBC count was 15.30 × 10^9^/L, and neutrophils were 94.80% on the 1st postoperative day. Moreover, on the 3rd day after the operation, his abdominal pain deteriorated and a full abdominal tenderness and rebound with muscle tension was noticed. In addition to the peritonitis signs, a bloody diarrhea occurred, and WBC count was as high as 15.26 × 10^9^/L and neutrophils were 84.1%. Enhanced CT scan showed thromboses in portomesenteric veins system. Meanwhile, a segmental small bowel wall thickening, edema, and ascites in the abdominal cavity were noticed (Figure 1B, C). A bloody ascites was acquired during diagnostic puncture, and the diagnoses of thrombophlebitis and intestinal infarction were established. The 2nd operation was performed on the postoperative day 3, during which a segmental necrosis of small bowel was found, and a bowel resection and anastomosis was preformed (Figure 2A). The thrombus in the portomesenteric veins was removed by using a Fogarty catheter from the edge of the mesentery (Figure 2B). The patient was transferred to the intensive care unit after the 2nd operation, and treated with systemic IV antibiotics of sulperazone (3.0 g, q8h) and metronidazole (0.5 g, q12h) and anticoagulation therapy of low-molecular-weight heparin (5700 IU, q12h for 3 days, then 3800 IU, q12h for 5 days), as well as a total parenteral nutrition support. The culture results from peripheral blood and thrombus were negative, and the ascites culture indicated the growth of *Escherichia coli*, thus the antibiotics was changed to Imipenem Cilastatin (0.5 g, q6h). The patient's condition recovered gradually after the 2nd operation, and an oral feeding started 11 days later, and then heparin infusion was replaced with warfarin (3 mg, qd). The WBC count and temperature were normalized on the 15th day after the 2nd operation.

Pathologic analysis showed a gangrenous appendicitis with local perforation for the appendix, without thrombus or abscess in the mesoappendix. Histologic analysis of the resected intestine indicated a transmural infarction, with thrombosis in the mesentery veins. The patient recovered and was discharged 22 days after the 2nd operation. Oral administration of warfarin continued for 3 months. A follow-up CT scan after 3 months showed no recurrence of portomesenteric veins thrombosis and the patient is in good condition (Figure 1D).

## DISCUSSION

Thrombophlebitis of the portomesenteric veins is a rare disease which is often related to intraperitoneal infections. The primary inflammatory diseases can be acute appendicitis, diverticulitis, Crohn disease, pancreatitis, cholecystitis, intraabdominal infection, and intrapelvic infection.^1^ Blood cultures could be positive in nearly 80% of the patients, and *Bacteroides fragilis*, *E coli*, *Proteus mirabilis*, *Clostridium species*, *Klebsiella*, *Pneumococcus*, *Aerobacter*, and *Streptococcus* have been reported as causative microorganisms.^1–3^ The incidence of pylephlebitis complicating appendicitis has become less since the advent of modern antibiotic therapy and aggressive surgical treatment of appendicitis.^1,3^ However, the reported mortality rate of pylephlebitis from all causes is still as high as 30 to 50%.^1,4^ This is partly because of the delay in the diagnosis due to nonspecific manifestation and a low index of suspicion, which might lead to intestinal infarction, liver abscess, and septic shock.

In our case, the thrombophlebitis of the portomesenteric veins was caused by a gangrenous appendicitis. There are several possible mechanisms of thrombogenesis in this condition: Thrombi are formed locally in the portomesenteric veins due to the hypercoagulability state caused by abscesses in the mesoappendix; Angiitis such as portal vein phlebitis occurs when the bacteria infiltrate into the portomesenteric veins; and Organisms invade into tissues around the veins to cause periphlebitis along the portomesenteric vessels, and when inflammation extends into the vascular lumens, thrombus forms.^4^ In our patient, thrombus in both the mesentery veins of small bowel and the portomesenteric veins were confirmed during the 2nd operation. However, the histological results of the resected appendix revealed neither thrombus in the mesenteria vessels nor local abscesses in the mesoappendix, and the culturing results of both blood and the IV thrombus were negative. We consider that the 3rd mechanism is the most possible reason for the thrombophlebitis in this case, because preoperative MSCT revealed no signs of portomesenteric veins thrombosis or bowel wall edema.

The initial manifestations of pylephlebitis include high fever, chills, malaise, right upper quadrant pain and tenderness, and occasional dysfunction of liver and fibrinolysis.^5^ These manifests are easy to be confused with the manifestations of the primary diseases, and lead to a low index of suspicion. The signs of peritonitis, bloody stool, and bloody ascites indicate the presence of bowel necrosis caused by intestinal ischemia.

Contrast-enhanced CT is the most effective diagnostic examination. The filling defect in the lumen of portomesenteric veins is observed in more than 90% of the cases.^1,6^ A sonography with color Doppler of the portomesenteric veins is also helpful for the diagnosis. However, these imaging modalities are not routinely performed for the diagnosis of acute appendicitis. Usually a nonenhanced CT scan is enough to make the diagnosis and assess the severity of the appendicitis in most patients, as in our case. The diagnosis of thrombophlebitis would not be difficult if a routine contrast-enhanced CT is introduced for the diagnosis of acute appendicitis.^4,6,7^ Chang et al^8^ reported 1 case of septic thrombosphlebitis of the portomesenteric veins 1 week after the resection of acute appendicitis, similar to our case. The reason of a late thrombosphlebitis complicating acute appendicitis after the resection in these 2 cases might be that organisms infiltrating around the mesenteric vessels are left after the operation, and a severe inflammation involving the vascular lumens leads to the formation of thrombus several days later.

Once the thrombophlebitis of the portomesenteric veins is suspected, appropriate treatments should be initiated as soon as possible. Traditional management includes IV antibiotics and long-term anticoagulation. Broad-spectrum antibiotics including antianaerobic agents are usually administered initially, and then replaced according to the culturing results from the blood or abdominal abscess. A minimum of 4 weeks of antibiotic therapy is recommended, and for patients with hepatic abscesses, the course of treatment is recommended to be at least 6 weeks.^7^ Despite the controversies, the administration of anticoagulants for at least 3 months is recommended in most cases.^9,10^ Normally subcutaneous injection or IV infusion of heparin is administered initially, and then shift to oral administration of warfarin.^11^ Surgical thrombectomy can make the blood vessels recanalized quickly, but high morbidity rate and risk of thrombosis relapse due to the damage of the local vascular wall has restrained its utility. For the majority of reported cases, long-term systematic antibiotics therapy and anticoagulants are efficient for the treatment of this disease.^10^

In this case, the patient developed a rapidly progressed thrombophlebitis of the portomesenteric veins which led to intestinal necrosis, even though the primary source of infection had been removed on the 1st onset day. This might be related to the severe inflammation around the vessels of portomesenteric system. However, a low index of suspicion may cause the delay of the diagnosis. If contrast-enhanced CT was performed on the 1st or 2nd day after appendectomy before the onset of peritonitis, the patients might be successfully treated by conservative managements, as reported previously.^8^ If an MSCT-angiography was performed before the 1st operation, the initial signs of portomesenteric veins thrombosis could be found, and the 2nd operation could be avoided. On the other hand, if a laparoscopic appendectomy was performed, the signs of early portomesenteric veins thrombosis or bowel wall edema may be noticed, with the advantage of laparoscopic approach to detect the entire abdominal cavity.

As differential diagnosis for this patient, a thrombophilia was considered and excluded. Several inherited causes such as factor V Leiden or prothrombin II mutations, dysfunction of antithrombin III and proteins S and C, and some other diseases such as myeloproliferative disease, antiphospholipid syndrome, and hyperhomocysteinemia, should be considered.^12^ In such conditions, anticoagulation should be administrated even longer, and the recurrence rate of thrombosis would be high. In our case, some factors including antithrombin, protein C activity, protein S activity, and anticardiolipin antibodies (ACA) had been tested after thrombectomy, and no signs of thrombophilia were found. Fortunately, our patient was successfully treated by the aggressive surgical procedures as well as the long-term administration of antibiotics and anticoagulants.

In summary, thrombophlebitis of the portomesenteric veins is a rare but fatal complication of acute appendicitis. It could occur even though the primary inflammatory appendix has been resected. For all the cases with acute abdominal pain, the possibility of thrombophlebitis should be considered as a differential diagnosis, and careful exanimation in each case will be very important. Once pylephlebitis is suspected, enhanced CT scan is helpful for early diagnosis, and sufficient control of inflammation as well as anticoagulant therapy should be performed as soon as possible.
